# Editorial: the changing faces of glutathione, a cellular protagonist

**DOI:** 10.3389/fphar.2015.00098

**Published:** 2015-05-15

**Authors:** Alfonso Pompella, Alessandro Corti

**Affiliations:** Department of Translational Research NTMC, University of PisaPisa, Italy

**Keywords:** glutathione, glutathione S-transferases, gamma-glutamyltransferase, oxidant stress, S-glutathionylation, redox regulation

Glutathione—the simple tripeptide composed of glutamic acid, cysteine, and glycine—is customarily defined as “*the most important intracellular non-enzymatic antioxidant*,” and most of the time such a swift definition is deemed sufficient to epitomize its functions in cellular homeostasis. But glutathione in fact is much more than that, as decades of passionate, unceasing research have documented. Just for instance, *extracellular* glutathione has also been shown to be of paramount importance in selected conditions—notably, respiratory pathophysiology—and functions of GSH other than antioxidant (indeed, even *prooxidant)* have been identified in years.

The title of the present e-Book explicitly recalls that of a fortunate commentary we wrote several years ago, back in 2003 (Pompella et al., 2003). During the decade or so since then, the interest in glutathione and related enzymes has continued to raise—slightly but steadily, as it had done in the decade preceding that publication (Figure 1). As also documented by the articles collected here, GSH biochemistry and pharmacology in fact involve wide and varied fields of interest in biology and medicine—from protein taxonomy to plant physiology, epigenetic regulation, cell energetics and the apoptosis/survival balance, carcinogenesis, drug resistance of cancer cells, inflammation-based diseases etc. It is thus very likely that experimental studies of diverse nature end up by encountering GSH at some earlier or later stage.

**Figure 1:**
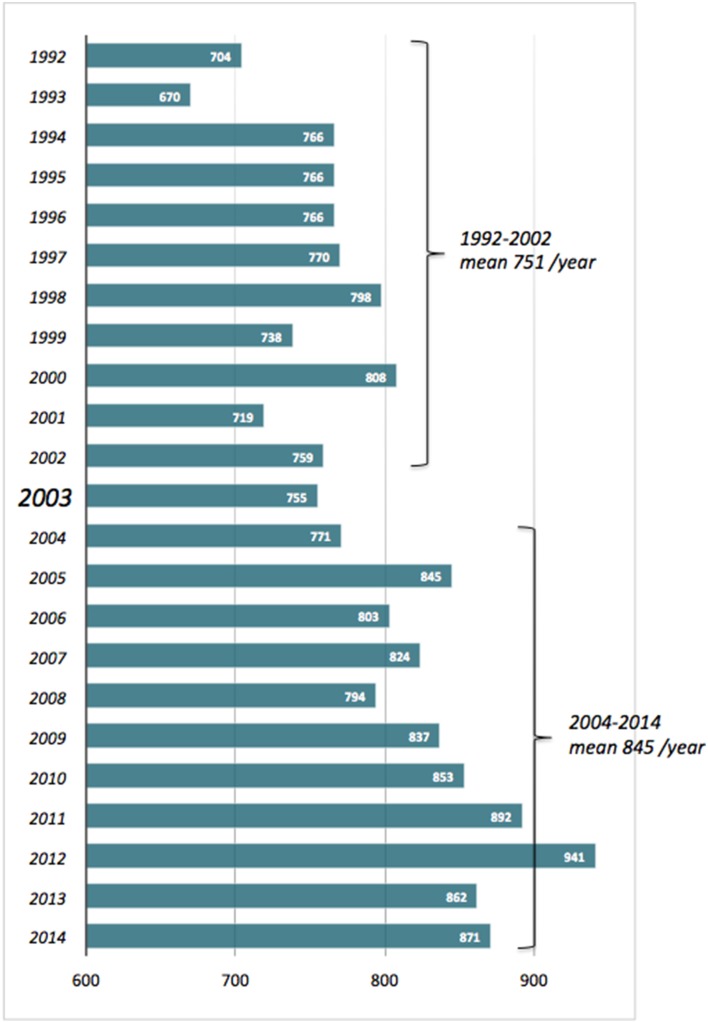
**No. of scientific papers published in the period 1992–2014 and quoting “glutathione” as title word**. Source: Entrez PubMed, http://www.ncbi.nlm.nih.gov/pubmed?cmd=Search.

The present collection of papers can be a useful tool in order to feel the pulse of current glutathione research in several fields of biological and medical interest. To start with, the first two articles in the series both offer—from distinct points of view—intriguing conceptual re-evaluations of GSH biochemical significance. First of all, the Opinion paper by Prof. L. Flohé's group provides punctual considerations pointing out that the electrochemical potential of GSH cannot entirely describe its redox regulating functions, and other kinetics and thermodynamic factors may be needed for a more comprehensive appraisal. Enzyme catalysis should be regarded as prioritary vs. thermodynamic or electrochemical parameters in GSH-dependent redox events; in short—the way these authors put it—“*redox regulation by GSH needs enzymes*” (Berndt et al., 2014). On the other hand, the article which follows is an admirably straightforward and clear Original research paper. Basing on the results of simple *in vitro* experiments with hypochlorous acid (HOCl), Profs. Haenen and Bast conclude that the partially oxidized forms of GSH generated in the GSH-HOCl reaction also are effective antioxidants, and do substantially contribute to the overall scavenging activity. Therefore the latter is best measured by the *total number of reactive molecules scavenged*, rather than by the reaction rate of the GSH-HOCl reaction (Haenen and Bast, 2014).

The two subsequent articles deal with structural and evolutionary aspects. The first one, an Original research paper, is an intriguing exploration of the factors which may have contributed to the selection of redox-active disulfides during the evolution of protein structures. S-glutathionylation, and the action of glutaredoxin as well as thioredoxin, are among these factors. The authors select three proteins for detailed analysis—CD4, ERO1, and AKT—and with the help of phylogenetic trees propose how redox-active disulfides may appear into a protein structure during evolution, by stepwise mutation of two residues in the native sequence to cysteine (Mohanasundaram et al., 2015). The following Review article by Lallement et al. is also focused on evolutionary aspects, and in particular on cysteine-containing glutathione transferases (Cys-GSTs) of plants. These peculiar enzymes can effect protein de-glutathionylation, thus mimicking the action of glutaredoxin; but, apart from the obvious function of dehydroascorbate reductases in regeneration of ascorbic acid, the functions of other Cys-GSTs in plants remain obscure. The authors try here to shed some light, and indeed do a precious job by including a phylogenetic analysis of the 14 classes of plant GSTs and a comprehensive survey of all aspects involved: gene expression, structure, catalytic mechanisms, subcellular localization, potential physiological roles (Lallement et al., 2014).

Epigenetics is the subject of the following two papers. The Opinion article by Garcia-Giménez and Pallardò highlights the roles played by GSH in gene regulation, as it directly binds to cysteine residues in H3 histone and it regulates the activity of S-adenosyl methionine synthetase (MAT1A), a major enzyme involved in DNA methylation. The authors thus propose that GSH is a factor linking epigenetic regulation with the redox status of the cell (García-Giménez and Pallardó, 2014). The subsequent Review is focused instead on the epigenetic regulation of glutathione S-transferase P1 (GSTP1), an ezyme activity also provided with “caretaker” function. Inactivation of this gene is often observed in human neoplasia (prostate, breast, and liver cancer, as well as leukemias), and is the result of acquired somatic CpG island promoter hypermethylation. The authors underscore the possibility of using such GSTP1 epigenetic alterations as biomarkers for early diagnosis (of prostate cancer in the first place), as well as potential targets of preventive or therapeutic treatments (Schnekenburger et al., 2014).

GSH of mitochondria plays a defensive role against reactive oxygen species deriving from respiration, and regulation of its levels impacts on the permeabilization of mitochondrial membrane, i.e., the first step in several instances of programmed cell death. This is the subject of the subsequent two Reviews and one Opinion article. The first paper (Ribas et al., 2014) includes a comprehensive appraisal of the involvement of mitochondrial GSH alterations in several important human pathologies, as well as a survey of the available strategies capable of restoring the mitochondrial pool of GSH and/or other antioxidants. The second Review evaluates the involvement of S-glutathionylation and/or thiols redox modulation in cell signaling related to nitric oxide induced responses, autophagic pathways and viral infection (Aquilano et al., 2014), while the Opinion paper which follows is an interesting reappraisal of studies showing how apoptosis can be linked to an intracellular oxidant stress consequent to active GSH extrusion, leading to formation of inter-protein disulfides implicated in the proapoptotic signaling. The authors note that GSH depletion may promote either pro-apoptotic or pro-survival pathways, depending on its kinetics of depletion, and speculate that the reactive cysteines of proteins involved in the opposite responses (e.g., cell-protective NF-kB component p50 vs. pro-apoptotic Bax) may be differently modulated according to the swiftness of the redox imbalance (De Nicola and Ghibelli, 2014).

The two subsequent Original research papers are dedicated to inflammation and immune-related diseases. The first one reports data confirming that erythrocytes of HIV patients show decreased GSH levels, an effect known to be due to HIV-1 transactivator protein (TAT) altering the expression of GSH biosynthetic enzymes (Choi et al., 2000). The authors conclude that resupplementing intracellular GSH by means of liposomal formulations may be effective in restoring immune functions, such as e.g., the antimycobacterial activity in macrophages from HIV patients (Morris et al., 2014). The other Original research paper in this subsection is concerned with an aspect of high relevance in respiratory medicine, i.e., the fundamental antioxidant and antiinflammatory role played by GSH contained in the epithelial lining fluid of airways. These levels are subject to the catabolizing activity of gamma-glutamyltransferase (GGT), an enzyme increasing in airways during inflammation. The authors elegantly demonstrate—in the IL-13 model of allergic airways inflammation in the mouse—that the pharmacological inhibition of airways GGT by means of a synthetic substrate analog, GGsTop (Han et al., 2007) results in a remarkable sparing of *extracellular GSH*, and can therefore augment antioxidant defenses and protect lung parenchyma against the prooxidant insults deriving from accumulation of activated inflammatory cells (Tuzova et al., 2014). The described approach might prove indeed valuable in lung diseases that perturb the extracellular lining fluid GSH pool, such as cystic fibrosis, acute respiratory distress syndrome (ARDS) and chronic obstructive pulmonary disease.

The conclusive three papers in our e-Book deal with cancer-related issues. The Review article by Prof. S. Toyokuni is a survey of the factors affecting the complex interplay of cellular transport systems for iron with those in charge of cellular supply of cysteines (Toyokuni, 2014). Excess iron is strictly linked with promotion of carcinogenesis, and on the other hand, the expression of the cystine/glutamate antiporter is intimately associated with ferroptosis, an iron-dependent, non-apoptotic form of cell death observed in cancer cells. The role of Nrf2/Keap1 signaling pathway—one of the most important cell defense and survival pathways—is discussed, as well as the recent finding that cancer stem cells in certain neoplasias present a stabilization of the xCT subunit of the x^–^_c_ cystine/glutamate antiporter, leading to increased intracellular GSH levels. This may explain the robustness of cancer cell defenses against oxidative stress, and is likely implicated in resistance to chemotherapeutic agents (Ishimoto et al., 2011).

The paper which follows also is a Review article, and focuses on the expression of glutathione S-transferases (GSTs) as well as of GGT as factors in the promotion of a more aggressive and resistant phenotype of cancer cells. GGT activity allows cells to maintain/reconstitute their GSH supplies which are utilized by GSTs for conjugation of xenobiotics, and in this way both enzyme activities synergistically contribute to detoxification of anticancer drugs. Nevertheless, pro-drugs have been synthesized that can be selectively *activated* by GSTs and GGT, whose expression is thus turned into a factor of *vulnerability* of the cancer cell. The Review recapitulates the different drug strategies involving GSH-conjugates and enzymes of the mercapturic acid pathway employed over the last two decades (Ramsay and Dilda, 2014). Amongst the long list of compounds investigated, a limited number of GSH-conjugates have recently progressed toward clinical trials, including both GST-activated nitrogen mustard TLK286 and GGT-activated arsenic-based prodrugs GSAO and Darinaparsin.

The very last paper of the e-Book also is the most speculative contribution to this series. In their Opinion paper Pennacchio et al. (2014) start from the observation that selected bacterial strains *(Helicobacter* spp. in the first place, plus others) can secrete active GGT enzyme, which causes depletion of GSH in cells of infected epithelia. The resulting oxidant stress has been put in connection with the invading ability of bacteria, i.e., with their virulence (Gong et al., 2010). A very similar phenomenon has been observed with selected parasitic insects, capable to secrete a GGT-containing venom capable of inducing GSH depletion in tissues of parasitized organisms. The authors highlight that several human neoplastic cell lines have also been shown to secrete active GGT enzyme, in the form of exosome-like vescicles (Franzini et al., 2009) resembling those secreted by *Helicobacter* (Zhang et al., 2013). It is thus proposed that the phenomenon may represent a true evolutionarily convergent strategy, spanning across very diverse life forms, aiming at depleting GSH levels in order to impair host's redox regulation and defenses to facilitate invasion and colonization. In this perspective cancer cells could be regarded as just another species of invasive “microrganisms,” and such a view appears consistent with other recent, unrelated observations pointing at the fact that cancer might conceivably be treated like an *infectious disease*, by even repurposing approved antibiotics for anticarcer therapy, across multiple tumor types (Lamb et al., 2015).

In summary, the articles collected in the present e-Book represent an assortment of updated data, hypotheses and interpretations deriving from a pretty wide spectrum of precincts in current biomedical research. This was somewhat expected, due to the exquisite interdisciplinarity of the subject, and in some way even desired, to fully exploit the informative potential typically owned by an online publication. Rather, as a matter of fact, it is felt that soon enough there might again be the need of a Topic and e-Book on glutathione—this seemingly inexhaustible *cellular protagonist*.

## Conflict of interest statement

The authors declare that the research was conducted in the absence of any commercial or financial relationships that could be construed as a potential conflict of interest.
